# Oxidative stress-induced apoptosis in granulosa cells involves JNK, p53 and Puma

**DOI:** 10.18632/oncotarget.15813

**Published:** 2017-02-28

**Authors:** Hongyan Yang, Yan Xie, Dongyu Yang, Decheng Ren

**Affiliations:** ^1^ Key Research Laboratory of Gynecology, Department of Gynecology, The Second Affiliated Hospital of Guangzhou University of Traditional Chinese Medicine, Yuexiu District, Guangzhou, Guangdong 510120, China; ^2^ Department of Medicine, The University of Chicago, Chicago, IL 60637, USA

**Keywords:** granulosa cell apoptosis, JNK, p53, Puma, H_2_O_2_

## Abstract

Reactive oxygen species (ROS) play important roles in follicular development and survival. Granulosa cell death is associated with increased ROS, but the mechanism of granulosa cell death induced by ROS is not clear. In order to define the molecular link between ROS and granulosa cell death, COV434, human granulosa tumor cells, were treated with H_2_O_2_. Compared to control cells, H_2_O_2_ induced granulosa cell death in a dose- and time-dependent manner. H_2_O_2_ induced an increase in Bax, Bak and Puma, and a decrease in anti-apoptotic molecules such as Bcl-2, Bcl-xL and Mcl-1. Both knockdown of Puma and overexpression of Bcl-xL could inhibit H_2_O_2_-induced granulosa cell death. These results suggest that suppression of Puma and overexpression of anti-apoptotic Bcl-2 family members could improve granulosa cell survival. To explore the mechanisms responsible for these findings, ROS in granulosa cells treatment with H_2_O_2_ were measured. The results showed that ROS was increased in a H_2_O_2_ dose- and time-dependent manner at the earlier time point. In addition, H_2_O_2_ induced an increase in Nrf2 and phosphorylation of JNK and p53. SP600125, an inhibitor of JNK, inhibits H_2_O_2_-induced phosphorylation of JNK and p53, and granulosa cell death. Antioxidant N-acetylcysteine (NAC) dose-dependently prevents H_2_O_2_-induced granulosa cell death. Furthermore, NAC also prevents phosphorylation of JNK and p53 induced by H_2_O_2_. Taken together, these data suggest that H_2_O_2_ regulates cell death in granulosa cells via the ROS-JNK-p53 pathway. These findings provide an improved understanding of the mechanisms underlying granulosa cell apoptosis, which could potentially be useful for future clinical applications.

## INTRODUCTION

Inside the follicular microenvironment, the oocyte is surrounded by several layers of granulosa cells that are differentiated into mural and cumulus granulosa cells during final stages of folliculogenesis. The morphology and number of encircling granulosa cells have been used as biomarkers for developmental competency, embryo and pregnancy outcome [1]. Granulosa cells not only provide nutrients and maturation-enabling factors to ensure successful maturation and developmental competency of oocytes but also protect oocytes from oxidative stress damage through their own antioxidant system during maturation of oocytes [2, 3]. Female reproduction aging is caused by ovarian aging with the core theory: the increase of follicles atresia rate reduces the number of ovarian follicles with age gradually. Granulosa cell apoptosis, a physiological phenomenon in follicle, can trigger follicular atresia and are responsible for follicle numbers decrease [4]. Thus the apoptosis of ovarian granulosa cells is also the main etiological factor of premature ovarian insufficiency. Regulation of granulosa cell apoptosis can be realized through the following two signaling pathways: “mitochondrial” or the intrinsic pathway and “death receptor” or the extrinsic pathway. Death receptor pathway is triggered by the combination of death receptor and the ligand, such as tumor related apoptosis inducing ligand (TRAIL) and its receptor, factor associated suicide (Fas) and its ligand (FasL). Mitochondrial pathway is mainly mediated by the Bcl-2 related proteins. The Bcl-2 family members such as Bid, Bim, Puma, Bax and Bak play a critical role in the intrinsic signaling pathway [5]. The relative contribution of extrinsic and intrinsic signaling pathways to granulosa cell apoptosis is unclear. Thus, clarifying the mechanisms responsible for ovarian granulosa cell apoptosis is important not only for understanding the pathogenesis of follicular atresia but also for developing novel approaches to prevent premature ovarian insufficiency.

Granulosa cells are sensitive to reactive oxygen species (ROS). ROS including H_2_O_2_ play a key role in granulosa cells apoptosis [6]. ROS, side-products formed during citric acid cycle, may cause oxidative damage through interacting with cellular lipids, proteins, and nucleic acids in cells. Increased ROS can induce oxidative stress when the balance between oxidation and reduction-regulated cellular processes is disrupted and cells cannot repair the resulting oxidative damage [7]. In human, oxidative stress may cause granulosa cell dysfunction [8]. Although endogenous H_2_O_2_ is an important signaling molecule, high levels of H_2_O_2_ may cause cell dysfunction and cell death. Exogenous H_2_O_2_ at concentrations ≥ 0.5 mM can rapidly induce cytotoxicity in human granulosa cell tumor line COV434 cells, which possess many characteristics of normal granulosa cells [9]. Increasing cellular antioxidant glutathione (GSH) can protect COV434 cells against H_2_O_2_-induced cytotoxicity. In contrast, depletion of the GSH sensitizes granulosa cells to toxicant-induced apoptosis [9]. In addition, generation of ROS caused by ionizing radiation and chemical toxicants has also been implicated in the toxicity of granulosa cells [10]. But the mechanism of cell toxicity induced by ROS is less known.

In the present study, COV434 cells were used to determine the mechanism of granulosa cell apoptosis induced by H_2_O_2_. The signaling pathways that mediate the effect of H_2_O_2_ in COV434 cells were also identified.

## RESULTS

### H_2_O_2_ induced granulosa cell death in a dose-and time-dependent manner

**Figure 1:**
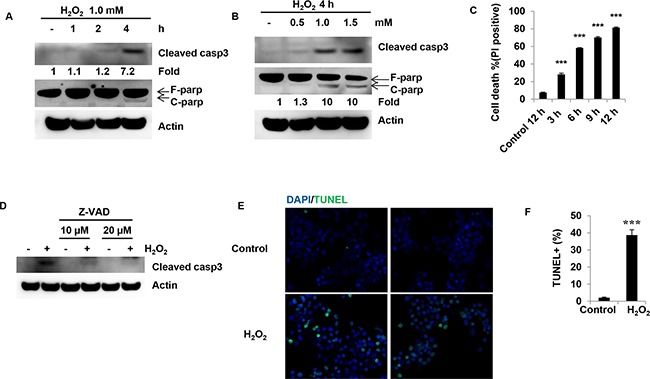
H_2_O_2_ induces cell death in human granulosa cells **(A)** Caspase3 and PARP protein levels in COV434 cells treated with H_2_O_2_ for different time. COV434 cells were treated with 1.0 mM H_2_O_2_ for a different time. Then cleaved caspase3 and PARP protein were assayed by western blot. The number depicts the relative changes in the levels of the indicated proteins using densitometry analysis of the Western blots in Figure 1A. **(B)** Caspase3 and PARP protein levels in COV434 cells treated with H_2_O_2_ at the different dose for 4 hours. COV434 cells were treated with H_2_O_2_ at 0.5, 1.0 and 1.5 mM for 4 hours. Then cleaved caspase3 and PARP protein were assayed by western blot. The number depicts the relative changes in the levels of the indicated proteins using densitometry analysis of the Western blots in Figure 1B. **(C)** Cell death was determined by PI-staining in COV434 cells. After treatment with 1.5 mM H_2_O_2_ for different time, the percentage of cell death was shown. **(D)** Z-VAD, caspase inhibitor, inhibits caspase3 cleavage. COV434 cells were treated with caspase inhibitor, 10 and 20 μM Z-VAD, for 2 hours prior to 1.0 mM H_2_O_2_ treatment for 4 hours. Cleaved caspase3 protein levels were determined by western blot. **(E)** TUNEL labeling of COV434 cells. 10 hours after 1.0 mM H_2_O_2_ treatment, apoptotic cells were assayed by TUNEL staining. **(F)** Quantitative TUNEL data are shown. ***P<0.001 compared to control group. Values are mean ± SEM.

H_2_O_2_ induced an increase in cleaved caspase3 protein levels in a time-dependent manner (Figure 1A). Treatment of COV434 cells with 1.0 mM H_2_O_2_ for 4 h induced a 6-fold increase in cleaved caspase3 (Figure 1A). H_2_O_2_ also time-dependently induced an increase in PARP cleavage (Figure 1A). In addition, H_2_O_2_ dose-dependently induced an increase in cleaved caspase3 and PARP (Figure 1B). After treatment of COV434 cells for 4 h, 1.5 mM H_2_O_2_ significantly induced an increase in cleaved PARP by 9-fold (Figure 1B). These results indicate that H_2_O_2_ induces ovarian granulosa cell death. Apoptotic ratios were analyzed by PI staining. Indeed, H_2_O_2_ at the concentration of 1.5 mM significantly induced cell death from 7.5% at 0 h to 58.2% at 6 h (Figure 1C). The highest apoptotic ratio was observed in COV434 cells at 12 h after H_2_O_2_ treatment (Figure 1C). Immunoblot analysis also showed that Z-VAD, caspase inhibitor, significantly inhibited the increase in cleaved caspase3 induced by H_2_O_2_ (Figure 1D). To further confirm that H_2_O_2_ treatment can cause cell apoptosis, another cell death assay, TUNEL staining, was used to measure cell apoptosis. The results showed that H_2_O_2_ significantly induced an increase in TUNEL positive staining from 2.1% in control cells to 38.7% in H_2_O_2_ treatment cells (Figure 1E and 1F), indicating more cells died after H_2_O_2_ treatment. These results suggest that H_2_O_2_ significantly induces COV434 cell death in both dose- and time-dependent manner.

### Bcl-2 family members are involved in H_2_O_2_-induced granulosa cell death

**Figure 2:**
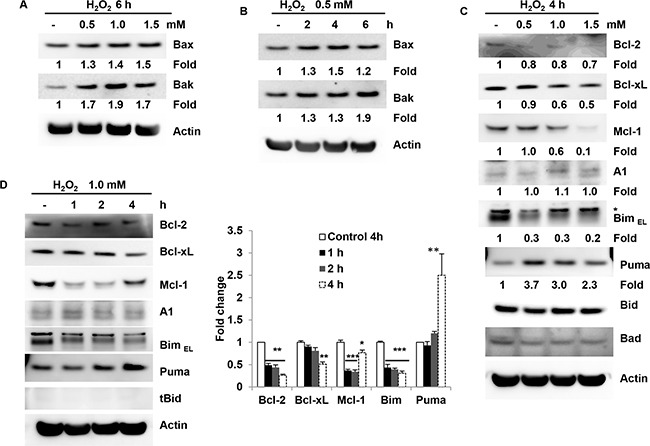
Bcl-2 family members are involved in H_2_O_2_-induced granulosa cell apoptosis **(A)** Western blot of Bax and Bak in COV434 cells. 6 hours after H_2_O_2_ treatment at 0.5, 1.0 and 1.5 mM in COV434 cells, immunoblot analysis was performed to determine Bax and Bak protein levels in COV434 cells. The number depicts the relative changes in the levels of the indicated proteins using densitometry analysis of the Western blots in Figure 2A. **(B)** Western blot of Bax and Bak in COV434 cells. After 0.5 mM H_2_O_2_ treatment for different time in COV434 cells, immunoblot analysis was performed to determine Bax and Bak protein levels in COV434 cells. The number depicts the relative changes in the levels of the indicated proteins using densitometry analysis of the Western blots in Figure 2B. **(C)** Western blot of Bcl-2 family members in H_2_O_2_- treated COV434 cells. 4 hours after H_2_O_2_ treatment at 0.5, 1.0 and 1.5 mM in COV434 cells, immunoblot analysis was performed to determine protein levels of Bcl-2 family members. The number depicts the relative changes in the levels of the indicated proteins using densitometry analysis of the Western blots in Figure 2C. **(D)** Western blot of Bcl-2 family members in H_2_O_2_-treated COV434 cells. After 1.0 mM H_2_O_2_ treatment for different time in COV434 cells, immunoblot analysis was performed to determine protein levels of Bcl-2 family members. The number depicts the relative changes in the levels of the indicated proteins using densitometry analysis of the Western blots in right of Figure 2. D. *P<0.05,**P<0.01,***P<0.001 compared to control group.

The Bcl-2 family has both pro-apoptotic members including Bid, Bim, Puma, Bax and Bak and anti-apoptotic members such as Bcl-2, Bcl-xL and Mcl-1. To determine whether H_2_O_2_ can change the expression levels of these molecules, we measured the protein levels of Bcl-2 family members. The results showed that H_2_O_2_ dose-dependently induced an increase in protein levels of Bax and Bak. H_2_O_2_ at 1.0 mM significantly increased Bax and Bak in COV434 cells at 6 h by 40% and 90%, respectively (Figure 2A). Furthermore, H_2_O_2_ also induced an increase in Bax and Bak in a dose-dependent manner. 0.5 mM H_2_O_2_ induced an increase in Bax protein levels by 40% at 4 h and Bak protein levels by 90% at 6 h (Figure 2B). Other Bcl-2 family members were also determined. H_2_O_2_ dose-dependently induced a decrease in Bcl-2, Bcl-xL and Mcl-1 and an increase in Puma at 4h (Figure 2C). 1.5 mM H_2_O_2_ induced a decrease in Bcl-xL and Mcl-1 protein levels at 4 h by 50% and 90%, respectively, and an increase 1.3-fold Puma levels. But the levels of A1, Bid and Bad were not changed by H_2_O_2_ treatment ingranulosa cells (Figure 2C). Surprisingly, Bim was decreased rather than increased after H_2_O_2_ treatment. Morevoer, H_2_O_2_ at 1.0 mM significantly induced a decrease in protein levels of Bcl-2, Bcl-xL, Bim and Mcl-1 and an increase in Puma in a time-dependent fashion (Figure 2D). However, the protein levels of A1 were unchanged and tBid was undetectable (Figure 2D). Interestingly, Mcl-1 was decreased after 1.0 mM H_2_O_2_ treatment at 2 and 4 h, but increased at 6h although it was not fully recovered. Taken together, these results suggest that H_2_O_2_ both dose-and time-dependently induces Bax, Bak and Puma up-regulation and Bcl-2, Bcl-xL and Mcl-1 down-regulation in COV434 cells.

### Puma contributes to H_2_O_2_-induced granulosa cell death

**Figure 3:**
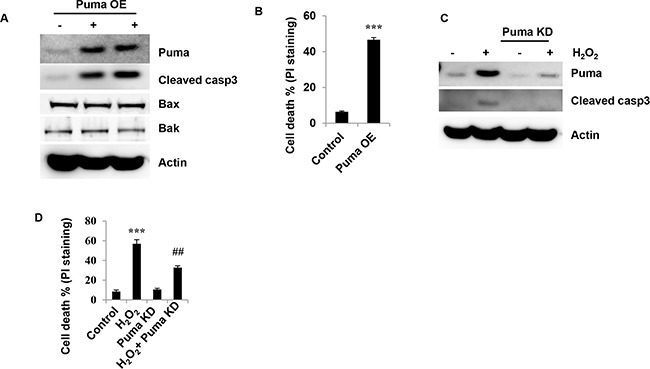
BH3-only molecule Puma mediates H_2_O_2_-induced granulosa cell death **(A)** Western blot of Puma in COV434 cells. 2 days after Puma overexpression (OE) in COV434 cells, immunoblot of cleaved caspase3 and Puma proteins in COV434 cells. **(B)** Cell death was determined by PI-staining in Puma OE COV434 cells. The percentage of cell death was shown. **(C)** Western blot of Puma and cleaved caspase3 in COV434 cells. 2 days after Puma knockdown (KD), COV434 cells were treated with 1.5 mM H_2_O_2_ for 6 hours. Then immunoblot analysis was performed to determine Puma and cleaved caspase3 protein levels in COV434 cells. **(D)** Measurement of Cell death. 2 days after Puma knockdown (KD), COV434 cells were treated with 1.5 mM H_2_O_2_ for 6 hours, cell death was determined by PI-staining (n=3). ***P<0.001 compared to control group. ##P<0.01 compared to H_2_O_2_ group.

To determine whether Puma up-regulation plays a role in granulosa cell death induced by H_2_O_2_, Puma was overexpressed by using a retroviral vector (Figure 3A). Overexpression (OE) of Puma dramatically increased the cleavage of caspase3 (Figure 3A). However, Bax and Bak protein levels were unchanged (Figure 3A). Consistent with this results, after 2 days infection, overexpression of Puma significantly increased cell death from 6.4% in control group to 46.6%, indicating Puma OE induces a 5.3-fold increase in cell death (Figure 3B). To further confirm the role of Puma in granulosa cell death, lentiviral shRNA was used to suppress Puma expression. Consistently, H_2_O_2_ increased Puma protein (Figure 3C). This effect was significantly inhibited by Puma knockdown (Figure 3C). Caspase3 activation was also inhibited in H_2_O_2_/Puma double knockdown (DKD) cells compared to H_2_O_2_ treatment alone (Figure 3D). Following Puma knockdown, 56.9 ± 4.2% of the cells were PI staining positive. In the H_2_O_2_/Puma DKD group, only 32.7 ± 1.8% (*P*<0.01 compared to H_2_O_2_ alone) took up the PI stain indicative of a 42% increase in cell viability (Figure 3D). Collectively, these findings show that Puma up-regulation contributes to apoptotic cell death induced by H_2_O_2_ in ovarian granulosa cells.

### Effect of Bcl-xL in H_2_O_2_-induced granulosa cell death

**Figure 4:**
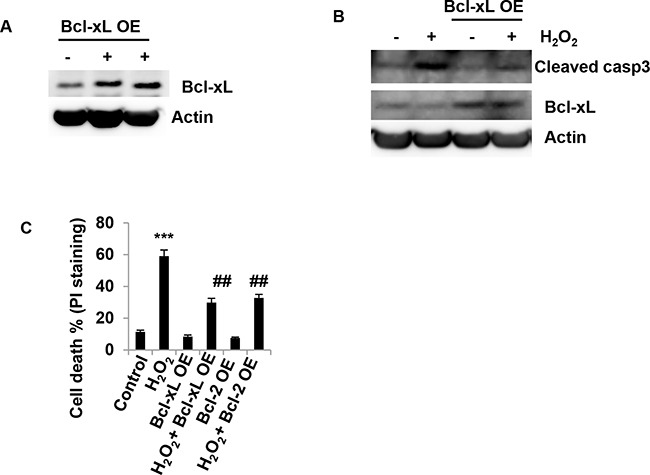
Overexpression of anti-apoptotic molecule Bcl-xL inhibits H_2_O_2_-induced granulosa cell death **(A)** Western blot of Bcl-xL in COV434 cells. 2 days after Bcl-xL overexpression (OE) in COV434 cells, immunoblot of Bcl-xL proteins in COV434 cells. **(B)** Western blot of Bcl-xL and cleaved caspase3 in COV434 cells. 2 days after Bcl-xL OE, COV434 cells were treated with 1.5 mM H_2_O_2_ for 6 hours. Then immunoblot analysis was performed to determine Bcl-xL and cleaved caspase3 protein levels in COV434 cells. **(C)** Measurement of Cell death. 2 days after Bcl-xL OE, COV434 cells were treated with 1.5 mM H_2_O_2_ for 6 hours, cell death was determined by PI-staining (n=3). ***P<0.001 compared to control group. ##P<0.01 compared to H_2_O_2_ group. Values are mean ± SEM.

The results described above showed that H_2_O_2_ induced a decrease in protein levels of anti-apoptotic molecules such as Bcl-2, Bcl-xL and Mcl-1. To determine whether increasing Bcl-xL expression can inhibits H_2_O_2_-induced cell death, Bcl-xL was overexpressed in COV434 cells (Figure 4A) The results showed that Bcl-xL OE prevented caspase3 activation and inhibited an increase in cleaved caspase3 protein induced by H_2_O_2_ treatment (Figure 4B). Bcl-xL OE also decreased H_2_O_2_-induced cell death. Following Bcl-xL OE, the PI stain positive cells decreased from 59.0 ± 3.8% in H_2_O_2_ treatment cells to 29.8 ± 2.7% (*P*<0.01) in H_2_O_2_/Bcl-xL OE cells on day 2 (Figure 4C). Bcl-2 OE also inhibited H_2_O_2_-induced cell death (Figure 4C).

### H_2_O_2_ induced intracellular ROS production in granulosa cells

**Figure 5:**
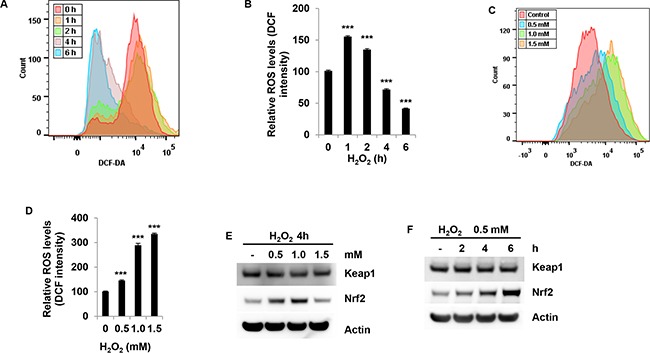
H_2_O_2_ induces the generation of intracellular ROS in granulosa cells **(A+B)** The effect of H_2_O_2_ on the intracellular ROS level was determined by flow cytometry analysis. COV434 cell were incubated with 1.5 mM H_2_O_2_ for different time, the intracellular ROS level was determined by flow cytometry analysis **(A)**. The results were shown as relative value after compared to control group of mean fluorescence **(B)**. **(C+D)** The effect of H_2_O_2_ on the intracellular ROS level was determined by flow cytometry analysis. COV434 cell were incubated with H_2_O_2_ at different concentration for 1 hour, the intracellular ROS level was determined by flow cytometry analysis **(C)**. The results were shown as relative value after compared to control group of mean fluorescence **(D)**. Western blot of Keap1 and Nrf2 in COV434 cells. 4 hours after H_2_O_2_ treatment at 0.5, 1.0 and 1.5 mM in COV434 cells **(E)** or the cells were treated with 0.5 mM H_2_O_2_ for different time **(F)**, immunoblot analysis was performed to determine Keap1 and Nrf2 protein levels in COV434 cells. ***P<0.001 compared to control group. Values are mean ± SEM.

To determine whether exogenous H_2_O_2_ increases intracellular ROS production, intracellular ROS production was measured by H2DCFDA. Indeed, H_2_O_2_ induced an increase in intracellular ROS production at 1 h in COV434 cells (Figure 5A). 1.5 mM H_2_O_2_ significantly increased ROS production by 50% at 1 h (P<0.001, Figure 5B). However, H_2_O_2_ significantly decreased ROS production after 1 h treatment (P<0.001, Figure 5A and 5B). For example, ROS production was decreased by 60% after H_2_O_2_ treatment at 6 h (P<0.001, Figure 5B). To further determine intracellular ROS production, COV434 cells were treated with different dose of H_2_O_2_ at 1 h. The results showed that H_2_O_2_ dose-dependently induced an increase in ROS production in cells (Figure 5C). 1.0 mM H_2_O_2_ significantly increased ROS production by 1.8-fold at 1 h (P<0.001, Figure 5D). Since the Keap1-Nrf2 pathway is the key regulator of protective responses to ROS, the Keap1 and Nrf2 protein levels in COV434 cells were determined after H_2_O_2_ treatment. After 4 h treatment, 0.5 and 1.0 mM H_2_O_2_ significantly increased Nrf2 protein levels but 1.5 mM H_2_O_2_ induced a decrease in its protein levels. Keap1 was only slightly decreased after 1.0 mM H_2_O_2_ treatment (Figure 5E). Moreover, 0.5 mM H_2_O_2_ time-dependently induced an increase in Nrf2 protein levels and has no effect on Keap1 (Figure 5F). Together with the above results, we conclude that H_2_O_2_ induces intracellular ROS production in a dose-dependent manner at the earlier stage, and lower dosage of H_2_O_2_ also induces an increase in Nrf2 protein.

### H_2_O_2_ induces JNK activation in granulosa cells

**Figure 6:**
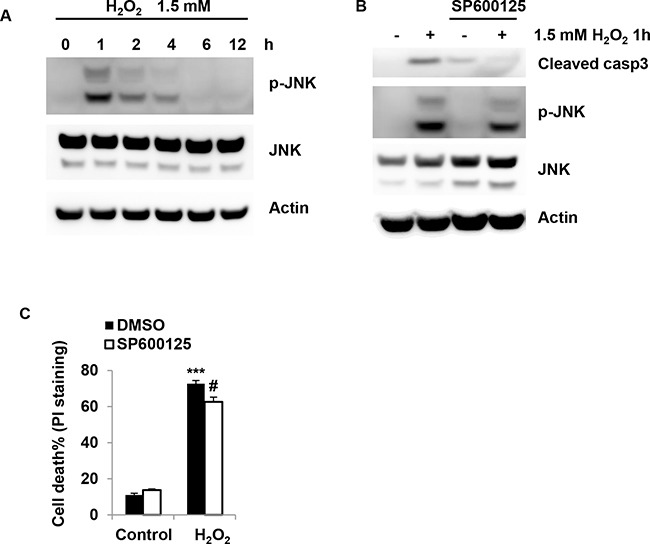
H_2_O_2_ induces H_2_O_2_ induces JNK activation in granulosa cells **(A)** Effect of H_2_O_2_ on the JNK in COV434 cells. COV434 cell were incubated with 1.5 mM H_2_O_2_ for different time, the phosphorylation of JNK was determined by western blot. **(B)** Effect of SP600125 on the JNK activation was determined by western blot. COV434 cell were incubated with 10 μM SP600125 for 3 hours, then 1.5 mM H_2_O_2_ was added to the medicum for 1 hour. JNK activation was determined by western blot. **(C)** Effect of SP600125 on the cell death induced by H_2_O_2_. COV434 cell were incubated with 10 μM SP600125 for 3 hours, then 1.5 mM H_2_O_2_ was added to the medicum for 6 hours. JNK activation was determined by western blot. Cell death was determined by PI-staining (n=3). ***P<0.001 compared to control group. #P<0.05 compared to H_2_O_2_ alone group.

To define the cell signaling pathway mediating the cell death induced by H_2_O_2_, JNK activation was determined in COV434 cells after H_2_O_2_ treatment. Phosphorylation of JNK was rapidly increased following treatment with 1.5 mM H_2_O_2_ for 1 h (Figure 6A). But after longer time treatment with H_2_O_2_, JNK activation was gradually deceased to baseline (Figure 6A). Then, SP600125, a JNK inhibitor, was used to determine the role of JNK in H_2_O_2_-induced cell death. The results showed that SP600125 inhibited phosphorylation of JNK and caspase3 activation (Figure 6B). Consistent with the results above, SP600125 at 10 μM significantly inhibited the granulosa cell death induced by H_2_O_2_ (P<0.05, Figure 6C). Collectively, these findings show that JNK is activated by H_2_O_2_ in granulosa cells.

### H_2_O_2_ triggers activation and accumulation of p53

**Figure 7:**
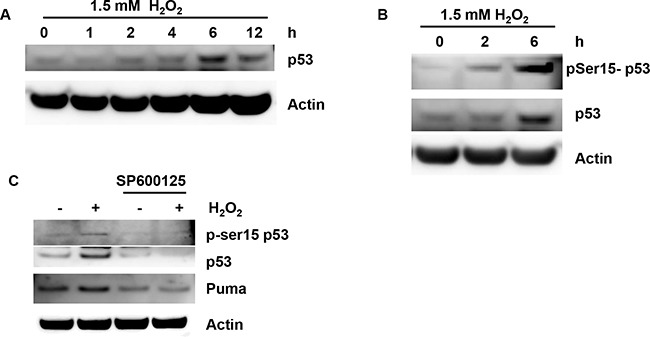
H_2_O_2_ induces p53 accumulation associated with JNK activation **(A)** Effect of H_2_O_2_ on the p53 in COV434 cells. COV434 cell were incubated with 1.5 mM H_2_O_2_ for different time, the protein lelves of p53 was determined by western blot. **(B)** Effect of H_2_O_2_ on the p53 activation in COV434 cells. COV434 cell were incubated with 1.5 mM H_2_O_2_ for 2 and 6 hours, the phosphorylatio of p53 and p53 were determined by western blot. **(C)** Effect of SP600125 on p53 activation and accumulation induced by H_2_O_2_. COV434 cell were incubated with 20 μM SP600125 for 3 hours, then 1.5 mM H_2_O_2_ was added to the medicum for 4 hours. p53 activation was determined by western blot.

We next determined the consequences of ROS production in COV434 cells with H_2_O_2_ treatment. ROS can cause DNA damage resulting in p53 activation. Thus p53 activation was determined in granulosa cells. Indeed, H_2_O_2_ at 1.5 mM induced the highest expression of p53 at 6 h (Figure 7A). Phosphorylation of p53 on Ser15 (pS15-p53) following 1.5 mM H_2_O_2_ treatment was in a time-dependent manner in COV434 cells (Figure 7B). JNK has been reported to be implicated in p53 accumulation [11]. Thus the effect of JNK on p53 was determined by JNK inhibitor. SP600125 inhibited phophorylation of p53 and p53 accumulation (Figure 7C). In addition, SP600125 also inhibited Puma up-regulation induced by H_2_O_2_ (Figure 7C). These results suggest that JNK initiates p53 accumulation and activation after H_2_O_2_ treatment in ovarian granulosa cells.

### Antioxidant NAC prevents generation of ROS induced by H_2_O_2_ in granulosa cells

**Figure 8:**
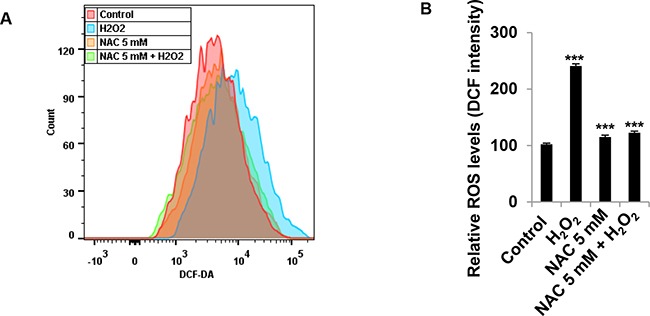
NAC prevents generation of ROS induced by H_2_O_2_ in granulosa cells **(A+B)** Effect of anti-oxidant NAC on the generation of ROS induced by H_2_O_2_ in COV434 cells. COV434 cell were incubated with 5 mM NAC for 1 hour prior to 1.5 mM H_2_O_2_ treatment for 1 hour. The intracellular ROS level was determined by flow cytometry analysis **(A)**. The results were shown as relative value after compared to control group of mean fluorescence **(B)**. ***P<0.001 compared to control group. Values are mean ± SEM.

Next, we addressed the question whether generation of ROS induced by H_2_O_2_ can be prevented by ROS scavenger *N*-Acetyl-L-cysteine (NAC). We found that antioxidant NAC at 5 mM prevented the generation of ROS induced by H_2_O_2_ (Figure 8A). Following NAC and H_2_O_2_ treatment, the generation of ROS decreased from 2.4-fold in H_2_O_2_ treatment cells to 1.2-fold (*P*<0.001) in NAC/H_2_O_2_ cells (Figure 8B). These results indicate that the generation of ROS is totally blocked by NAC.

### Antioxidant NAC prevents ROS-mediated JNK activation and p53 accumulation in granulosa cells

**Figure 9:**
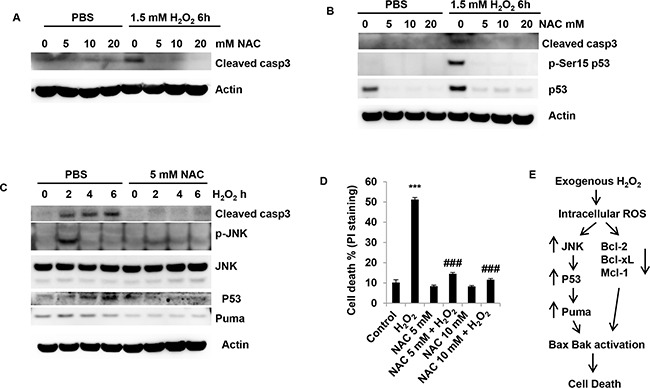
NAC prevents ROS-mediated JNK activation and p53 accumulation in granulosa cells **(A)** Effect of anti-oxidant NAC on caspase3 cleavage induced by H_2_O_2_ in COV434 cells. COV434 cell were incubated with NAC at different concentration for 1 hour prior to 1.5 mM H_2_O_2_ treatment for 6 hours. The cleaved capase3 was determined by western blot. **(B)** Effect of anti-oxidant NAC on p53 activation and accumulation induced by H_2_O_2_ in COV434 cells. COV434 cell were incubated with NAC at different concentration for 1 hour prior to 1.5 mM H_2_O_2_ treatment for 6 hours. The phosphorylation and accumulation of p53 were determined by western blot. **(C)** Effect of anti-oxidant NAC on JNK activation and p53 accumulation induced by H_2_O_2_ in COV434 cells. COV434 cell were incubated with 5 mM NAC for 1 hour prior to 1.0 mM H_2_O_2_ treatment for different time. The phosphorylation of JNK, p53 accumulationand Puma protein levels were determined by western blot. **(D)** Effect of anti-oxidant NAC on cell death induced by H_2_O_2_ in COV434 cells. COV434 cell were incubated with 5 mM NAC for 1 hour prior to 1.5 mM H_2_O_2_ treatment for 6 hours. Cell death was determined by PI-staining (n=3). ***P<0.001 compared to control group. ###P<0.001 compared to H_2_O_2_ alone group. **(E)** Model of H_2_O_2_-induced granulosa cell death. Exogenous H_2_O_2_ increases the generation of intracellular ROS in granulosa cells. The accumulation of ROS leads to activation of JNK. Activated JNK facilitatses p53 activation and accumulation. p53 upregulates Puma, which induces Bax and Bak activation and leads to the robust apoptosis. ROS - induced suppression of Bcl-2, Bcl-xL and Mcl-1 also contribute to apoptosis induction.

The above results demonstrate that JNK and p53 are involved in H_2_O_2_-induced cell death. Thus, we speculated that NAC might prevent a ROS-JNK-p53 cycle to protect granulosa cells from cell death triggered by H_2_O_2_. The results showed that even at 5 mM NAC efficiently prevented caspase3 activation (Figure 9A and 9B). NAC at 5 mM also obviously reduced phosphorylation of p53 and p53 accumulation, indicating that ROS was involved in p53 accumulation (Figure 9B). Consistently, NAC obviously reversed the activation of JNK and inhibited Puma up-regulation (Figure 9C), suggesting that JNK was a critical player downstream of ROS in the signal pathway leading to p53 activation. Moreover, NAC fully rescued the cell death induced by H_2_O_2_ (Figure 9D). The cell death decreased from 51% in H_2_O_2_ treatment cells to 14.5% in NAC/H_2_O_2_ treatment cells (P<0.001, Figure 9D). Figure 9E shows the model of H_2_O_2_-inducedgranulosa cell death. Exogenous H_2_O_2_ increases the generation of intracellular ROS in granulosa cells. The accumulation of ROS leads to activation of JNK. Activated JNK facilitatses p53 activation and accumulation. p53 upregulates Puma, which induces Bax and Bak activation and leads to the robust apoptosis. Suppression of Bcl-2, Bcl-xL and Mcl-1 also results in apoptosis.

All these results demonstrated that ROS-JNK-p53 pathway plays a key role in regulating cell death in H_2_O_2_-treated COV434 cells, and antioxidant NAC prevents ROS-mediated JNK activation and p53 accumulation in ovarian granulosa cells.

## DISCUSSION

Preovulatory as well as ovulated oocytes are encircled with several layers of cumulus and mural granulosa cells which constitute the largest group of cells in the ovary and play a crucial role in follicle development and oocyte competence. Physiologic function of granulosa cell is dependent on paracrine and autocrine cytokines in ovarian microenvironment and reproductive hormones in peripheral blood [12]. The reduced number of granulosa cells and disruption in the cell-cell communication might have deprived oocyte of nutrients and survival factors inside the preovulatory follicle and induced apoptosis in ovulated oocytes. Human ovaries produce a constant number of oocytes during every menstrual cycle. However, the majority of follicles undergo atresia, which is mediated by apoptosis [13]. A progressive reduction in granulosa cells is a key element in the development of antral follicle atresia. The apoptosis of granulosa cells leads to follicular atresia due to the insufficiency of survival signals and/or physiologic/nonphysiologic apoptotic signals [14].

Apoptosis can be triggered by various stimuli from outside or inside the cells. It has also been suggested that the age-related decline in oocyte quality and fertility is modulated by oxidative stress [15]. Oxidative stress also contributes to granulosa cell apoptosis [16]. Therefore protecting granulosa cells against oxidative stress-induced apoptosis might be of great therapeutic value in the treatment of reproductive ageing. Data of the present study suggest that H_2_O_2_ increased the granulosa cell death in a dose- and time-dependent manner. Caspase3 activation, PARP cleavage and TUNEL positive staining were increased after H_2_O_2_ treatment. Z-VAD almost completely inhibited caspase3 cleavage. These results suggest that H_2_O_2_ can induce apoptosis in ovarian granulosa cells, which was consistent with the previous studies [17]. Since Bcl-2 family proteins are the major regulators of apoptosis, we determine how Bcl-2 family members are involved in granulosa cell apoptosis induced by H_2_O_2_. The results showed that H_2_O_2_ decreased anti-apoptotic molecules Bcl-2, Bcl-xL and Mcl-1, and increased pro-apoptotic molecules such as Bax, Bak and Puma. To further confirm that Puma was a player in granulosa cell apoptosis, the effect of overexpression of Puma on granulosa cells was determined. Indeed, the results showed that overexpression of Puma induced COV434 cell apoptosis. In contrast, Puma KD significantly decreased the cell apoptosis induced by H_2_O_2_. On the other hand, overexpression of the Bcl-xL inhibited H_2_O_2_-induced granulosa cell apoptosis. Due to the BH3-only molecule Puma directly inducing a stepwise activation of Bax and Bak, increasing Puma would results in granulosa cell apoptosis. Furthermore, autoactivation of Bax and Bak can occur independently of activator BH3s through downregulation of Bcl-2, Bcl-xL and Mcl-1 [18]. Therefore, H_2_O_2_-induced granulosa cell apoptosis was mediated by Bax and Bak activation through both increasing Puma and decreasing anti-apoptotic molecules Bcl-2, Bcl-xL and Mcl-1.

Although ROS play a role in many cellular processes, high levels of ROS can induce cellular damage such as DNA damage, polyunsaturated fatty acids and amino acids oxidations, and specific enzymes deactivation by oxidation of co-factors. To further identify the mechanisms of granulosa cell death induced by H_2_O_2_,we determined the generation of intracellular ROS. Exogenous H_2_O_2_ dose-dependently increased the generation of intracellular ROS at the earlier time point. After 4 hours treatment with exogenous H_2_O_2_,the generation of ROS was reduced. One explanation is that higher ROS generated at the earlier time damages the cell membrane, which leads to the leak of fluorescent DCF. Thus fluorescence is lower at later time after H_2_O_2_ treatment. Another explanation is that cells use defense mechanisms to ameliorate the ROS. Indeed, here we showed that after H_2_O_2_ treatment, although Keap1 only slightly decreased, Nrf2 significantly increased. Keap1 serves as the substrate adaptor subunit in the E3 holoenzyme in the ubiquitination pathway, leading to Nrf2 ubiquitination and degradation. H_2_O_2_ can cause Keap1 oxidation, leading to a conformational change of Keap1. Such conformational alterations inhibit the binding between Nrf2 and Keap1, thus stopping Nrf2 ubiquitination and degradation [19]. Ultimately, Nrf2 translocates to the nuclear and regulates the expressions of antioxidants [20]. Therefore we assume that the increase in Nrf2 is the protective response of granulosa cells to ROS-induced stress. Moreover, in the present study, anti-oxidant NAC efficiently protects COV434 cells against cell death induced by ROS. As a synthetic precursor of intracellular cysteine and glutathione, the anti-ROS activity of NAC results from its free radical scavenging property either directly via the redox potential of thiols, or secondarily via increasing glutathione levels in the cells [21].

It has been well established that JNK, a member of the mitogen-activated protein kinases(MAPKs) pathway, plays a critical role in both extrinsic and intrinsic apoptotic pathway [22]. To determine the mechanism by which increased ROS cause cell death, the effect of JNK on cell death was investigated. Here we found that H_2_O_2_ induced JNK phosphorylation (p-JNK) at the earlier time point, which was consistent with the increased ROS levels at that time. Importantly, p-JNK induction was due to the increased ROS, since NAC inhibited JNK activation. Notably, JNK serves as a critical mediator of the H_2_O_2_-induced apoptosis, as also evidenced by the inhibition of caspase3 cleavage and cell death by JNK inhibitor SP600125.

In response to DNA damage induced by oxidative stress, wild-type p53 orchestrates transcriptions of numerous genes and induces cells either to cell cycle arrest, senescence, or apoptosis via differential activation of target genes, preventing the propagation of damaged DNA [23]. Our studies showed that H_2_O_2_ induced an increase in phosphorylation of p53 and p53 accumulation. JNK inhibitor SP600125 inhibited the activation and accumulation of p53, indicating that p53 induction is due to JNK activation. p53 can also trigger cell apoptosis by directly activating Bax or by upregulating Puma to indirectly activate Bax and Bak. On the other hand, NAC fully reversed the apoptosis triggered by H_2_O_2_ and inhibited the activation of JNK and p53 accumulation. These data further confirmed that ROS was involved in the activation of JNK and accumulation of p53.

In conclusion, exogenous H_2_O_2_ dose- and time-dependently increased granulosa cell apoptosis which related to inducing a decrease in anti-apoptotic members such as Bcl-2, Bcl-xL and Mcl-1, and an increase in BH3-only molecule Puma. Exogenous H_2_O_2_ dose-dependently increased the generation of intracellular ROS at the earlier time point. H_2_O_2_ increases Nrf2 and promotes the activation of JNK and p53 accumulation via generation of ROS. Anti-oxidant NAC prevented H_2_O_2_-induced cell death by deactivating JNK and inhibiting p53 accumulation through inhibiting ROS production. These results suggest antioxidant therapeutic interventions may have beneficial effects on follicular atresia associated with ovarian granulosa cell apoptosis.

## MATERIALS AND METHODS

### COV434 cell culture

COV434 cells were purchased from Sigma and maintained in Dulbecco’s modified Eagle’s medium–F12 (DMEM–F12) with GlutaMAX™ supplemented with 9% FBS, henceforth referred to as complete media, at 37°C in a humidified atmosphere of 95% air and 5% CO_2_. During maintenance, cells were passaged every 4–5 days. Cells from passages 25–50 were used for the experiments reported herein. For experiments, cells were harvested by trypsinization, counted using a hemacytometer, and were plated in complete medium. For protein extraction COV434 cells were plated 5 × 10^6^ cells/flask or 2× 10^5^ cells/well of 6-well plate for H_2_O_2_ treatment. Cells were plated 5 × 10^4^ cells per chamber of eight-chamber Lab-Tek II CC2 tissue culture slides for TUNEL and Hoechst 33342 staining.

### Quantitation of cell death

Cell death was quantified by propidium iodide staining [24], followed by flow cytometric analyses using a FACS Caliber (BD Bioscience) and FlowJo software. Propidium iodide (PI) intercalates into double-stranded nucleic acids. PI is excluded by viable cells but can penetrate cell membranes of dying or dead cells. 20 μM pan-caspase inhibitor benzyloxycarbonyl-Val-Ala-Asp-fluoromethylketone (Z-VAD) was added to the medium 2 hours prior to treatment COV434 cells by H_2_O_2_. Z-VAD was added to the cells on day1.

### Retrovirus infection

Mouse Bim_EL_ and Puma were cloned into the retroviral expression vector MSCV-IRES-GFP (pMIG) (Clontech). The production of amphotropic retroviruses using the 293GPG packing cell line was performed as described previously [25]. Retroviral plasmids were transfected using X-tremeGene 9 DNA transfection reagent (Roche) according to the manufacturer’s protocols. Retroviral transduction of each indicated BCL-2 family protein was confirmed by western blotting. Two days after infection with Bcl-xL retrovirus, COV434 cells were lysed and probed with antibody for immunoblot analysis.

### Lentivirus-mediated shRNA expression

The pLKO.1-puro lentivirus vector was generously provided by Dr. Sheila Stewart of Washington University Medical School (St. Louis, MO). Lentiviral shRNA targets in the murine Puma mRNA were purchased from GE Dharmacon (RMM4534) and identified using western blot. Recombinant lentiviral particles were prepared by transfecting HEK 293T cells with the appropriate pLKO.1-puro plasmid plus pHR′CVM8.2 delta R and pCMV-VSV-G plasmids. Lentivirus was added to the medium on day 1.

### Western blot

COV434 cells and islets were lysed in RIPA cell lysis buffer (Thermal Scientific) containing PMSF, Na_3_VO_4_, and complete protease inhibitor mixture. Equal amounts of protein were resolved by 10% or 4–12% NuPAGE (Invitrogen) gels, transferred onto PVDF membrane (Immobilon-P; Millipore), and blots were probed with antibodies against Bax (N20) (SC493, Santa Cruz) and Bak (06536, Upstate), Bcl-2 (554218; Pharmigen), Bcl-xL(2762, Cell Signaling), Puma (7467; Cell Signaling Technologies), Bim (202000; Calbiochem), cleaved caspase3 (9661; Cell Signaling Technologies), phospho-JNK (Thr183/Tyr185) (4668; Cell Signaling), JNK (9258; Cell Signaling), phospho-p53 (Ser15)(12571; Cell Signaling), p53(2524; Cell Signaling), actin (A-2066; Sigma). Antibody detection was accomplished using enhanced chemiluminescence method (Western Lightning, PerkinElmer) and LAS-3000 Imaging system (FUJIFILM).

### Detection of intracellular ROS [26]

The intracellular levels of ROS were measured by loading the COV434 cells with the fluoroprobe H2-dichlorofluorescein diacetate (H2DCF-DA) (Invitrogen). Dichlorofluorescein diacetate (DCF-DA) is taken up by cells, is cleaved to DCF by intracellular esterases, and is oxidized in the presence of ROS to DCF. The H2DCF-DA probe was freshly reconstituted in DMSO before loading. In brief, cells were cultured 5 × 10^4^ cells per well of 12-well plate in 1ml of complete medium for 24 h. For measurement of the effect of H_2_O_2_ treatment on ROS generation, the wells were washed with PBS after H_2_O_2_ treatment, loaded for 30 min at 37°C with 1μM DCF-DA in medium. Then the DCF-DA containing medium was removed, cells were washed with PBS, and maintained in PBS while fluorescence intensity was read by fluorescence-activated cell sorting (FACS). The fluorescence intensity was analyzed and quantified using Flowjo. The results are representative of 3 independent cultures.

### TUNEL staining

The terminal deoxynucleotidyltransferase-mediated dUTP nick end labeling (TUNEL) labeling used the Dead End Fluorometric TUNEL System (Promega Corp). TUNEL technique was used to detect DNA strand breaks formed during apoptosis. More than 300 cells were counted after TUNEL staining and at least 3 wells were counted per group.

### Statistical analysis

The 2-tailed unpaired Student’s *t* test was used to assess the statistical significance of differences between 2 sets of data. Differences were considered significant when *P* < 0.05. In all experiments, the number of asterisks is used to designate the following levels of statistical significance: *** p < 0.001, **p < 0.01, *p < 0.05 compared with control group. Results are presented as mean ± SEM.
